# Caveolin-1 impairs PKA-DRP1-mediated remodelling of ER–mitochondria communication during the early phase of ER stress

**DOI:** 10.1038/s41418-018-0197-1

**Published:** 2018-09-12

**Authors:** Roberto Bravo-Sagua, Valentina Parra, Carolina Ortiz-Sandoval, Mario Navarro-Marquez, Andrea E. Rodríguez, Natalia Diaz-Valdivia, Carlos Sanhueza, Camila Lopez-Crisosto, Nasser Tahbaz, Beverly A. Rothermel, Joseph A. Hill, Mariana Cifuentes, Thomas Simmen, Andrew F. G. Quest, Sergio Lavandero

**Affiliations:** 1 Advanced Center for Chronic Diseases (ACCDiS), Facultad de Ciencias Químicas y Farmacéuticas & Facultad de Medicina, Universidad de Chile, 8380492 Santiago, Chile; 2 Instituto de Nutrición y Tecnología de los Alimentos (INTA), Universidad de Chile, 7830490 Santiago, Chile; 3 Center for Exercise, Metabolism and Cancer (CEMC), Facultad de Medicina, Universidad de Chile, 8380492 Santiago, Chile; 4 Department of Cell Biology, University of Alberta, Edmonton, AB T6G 2H7 Canada; 5 Cardiology Division, Department of Internal Medicine, University of Texas Southwestern Medical Center, Dallas, TX 75390 USA

**Keywords:** Cell biology, Cancer

## Abstract

Close contacts between endoplasmic reticulum and mitochondria enable reciprocal Ca^2+^ exchange, a key mechanism in the regulation of mitochondrial bioenergetics. During the early phase of endoplasmic reticulum stress, this inter-organellar communication increases as an adaptive mechanism to ensure cell survival. The signalling pathways governing this response, however, have not been characterized. Here we show that caveolin-1 localizes to the endoplasmic reticulum–mitochondria interface, where it impairs the remodelling of endoplasmic reticulum–mitochondria contacts, quenching Ca^2+^ transfer and rendering mitochondrial bioenergetics unresponsive to endoplasmic reticulum stress. Protein kinase A, in contrast, promotes endoplasmic reticulum and mitochondria remodelling and communication during endoplasmic reticulum stress to promote organelle dynamics and Ca^2+^ transfer as well as enhance mitochondrial bioenergetics during the adaptive response. Importantly, caveolin-1 expression reduces protein kinase A signalling, as evidenced by impaired phosphorylation and alterations in organelle distribution of the GTPase dynamin-related protein 1, thereby enhancing cell death in response to endoplasmic reticulum stress. In conclusion, caveolin-1 precludes stress-induced protein kinase A-dependent remodelling of endoplasmic reticulum–mitochondria communication.

## Introduction

Communication between the endoplasmic reticulum (ER) and mitochondria is essential to coordinate cellular responses [1, 2]. Both organelles form contact points via cholesterol-rich microdomains, termed mitochondria-associated ER membranes (MAM) [3], which allow for efficient Ca^2+^ transfer from ER to mitochondria [4] and either stimulate mitochondrial bioenergetics [5] or initiate apoptosis [6]. Previously, we showed that disruption of the ER protein folding capacity, termed ER stress, during its early stage increases the ER–mitochondria contacts, thus leading to an adaptive increase in mitochondrial ATP production [7]. This appears to be a generic response to acute stress, as we also observed such changes upon inhibition of the nutrient-sensing kinase mammalian target of rapamycin complex 1 (mTORC1) [8]. On the other hand, alterations in ER–mitochondria contacts have also been reported in various models of chronic disease [9–12].

Among the regulators of the ER–mitochondria interface, Calnexin is an ER-resident chaperone that regulates the Ca^2+^-handling machinery at MAM. Upon ER stress, Calnexin translocates from MAM to the ER, fulfilling a dual objective: (a) to reinforce protein folding at the ER, and (b) to enhance ER–mitochondria Ca^2+^ transfer [13]. Therefore, MAM composition is dynamic and requires appropriate membrane organization.

Caveolin-1 (CAV1) is a scaffolding protein that controls intracellular cholesterol transport [14] and numerous processes related to cell death and survival at the plasma membrane [15]. A recent report showed that CAV1 is enriched at MAM, and its ablation greatly reduces ER–mitochondria contact sites while increasing inter-organelle cholesterol transfer [16]. These observations agree with previous studies showing a requirement for CAV1 presence in lipid raft-like domains of the ER [17] to protect against mitochondrial dysfunction induced by cholesterol overload [18]. Nonetheless, it remains unclear how CAV1 impacts on signalling cascades that regulate organelle communication.

Regarding the potential pathways governing ER–mitochondria interaction, ER stress and mTORC1 inhibition share in common the activation of cAMP-dependent protein kinase (PKA). mTORC1 inhibition leads to PKA-mediated inhibitory phosphorylation of Dynamin-related protein-1 (DRP1) on Ser637 [19]. DRP1 is a GTPase that orchestrates mitochondrial fragmentation by forming a constrictive ring around mitochondria, and thus its inhibition by PKA promotes mitochondrial elongation. Of note, DRP1-mediated fragmentation occurs at the ER–mitochondria interface [20] and is regulated by ER-localized proteins [21, 22]. Similarly, ER stress also leads to PKA activation as a protective mechanism [23], which is partially due to DRP1 phosphorylation [24]. Interestingly, DRP1 phosphorylation at Ser637 upon ER stress has been associated with its translocation to the ER, where it participates in ER expansion triggered to cope with protein misfolding [25]. Therefore, PKA regulates the dynamics of both organelles by determining DRP1 distribution and function [26]. Interestingly, CAV1 and CAV3 reportedly serve as PKA-anchoring proteins on the surface of lipid droplets in adipocytes [27] and in T-tubules in cardiomyocytes [28], respectively. However, whether CAV1 modulates the PKA-DRP1 axis required for the regulation of ER–mitochondria communication remains unexplored.

In light of these observations, we hypothesized that CAV1 regulates ER–mitochondria interaction during early ER stress by modulating PKA-mediated DRP1 phosphorylation at MAM. Consistent with this hypothesis, we report that the PKA-DRP1 pathway is required to enhance ER–mitochondria communication during early ER stress. CAV1, in turn, is present at MAM, and blocks PKA-induced DRP1 translocation required for cell survival. Thus CAV1 reduces ER–mitochondria communication and thereby increases sensitivity to ER stress.

## Results

### CAV1 antagonizes ER–mitochondria communication

**Fig. 1:**
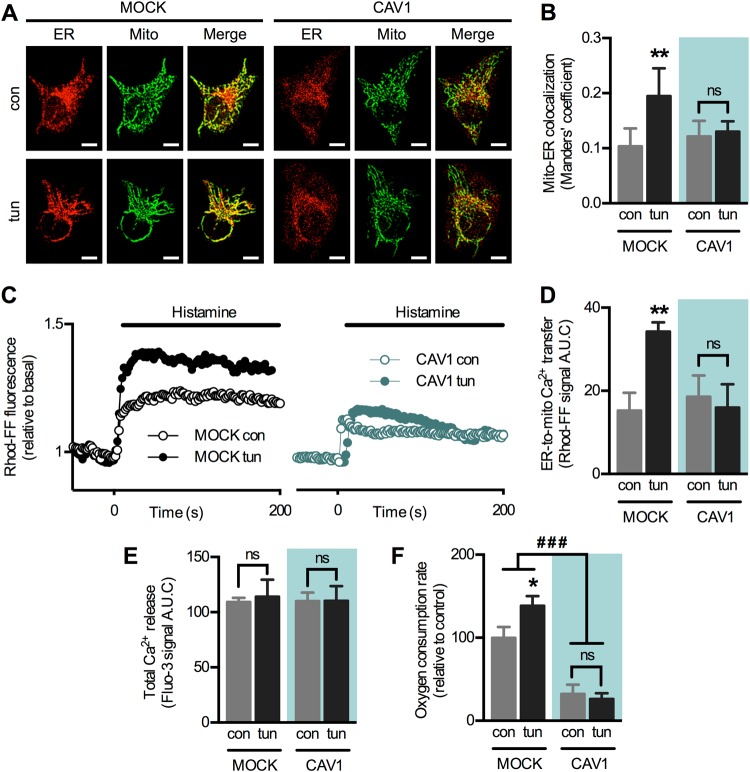
Caveolin-1 expression reduces ER–mitochondria communication during the early phase of ER stress in HeLa cells. **a** HeLa mock and CAV1 cells in control condition (con) or under early ER stress with tunicamycin (tun) were processed for live-cell imaging. The ER was stained with ER-Tracker Red and mitochondria were stained with MitoTracker Green and then imaged using confocal microscopy. **b** Mitochondria–ER colocalization was quantified as Manders’ coefficients of images obtained in **a** (*n* = 3). **c** For experimental groups as in **a**, Ca^2+^ release from ER stores was induced with histamine, while mitochondrial Ca^2+^ levels were imaged with Rhod-FF using fluorescence microscopy. **d** Quantification of the area under the curve (A.U.C.) of graphs obtained in **c** (*n* = 3). **e** For cells treated as in **c**, Ca^2+^ release from ER stores was imaged with Fluo-3 using fluorescence microscopy and the resulting A.U.C. was quantified (*n* = 3). **f** For experimental groups as in **a**, mitochondrial respiration rates were measured using a Clark electrode (*n* = 3). For each independent imaging experiment, 5–15 cells were analysed. Scale bars: 10 µm. Results are shown as mean ± s.e.m. *P < 0.05 and **P < 0.01 compared with respective con condition. ^###^*P* < 0.001 overall comparison between mock and CAV1 cells. ns non-significant

First, we examined whether CAV1 alters the remodelling of ER–mitochondria coupling during early ER stress. For these studies we chose HeLa cells, which express low levels of endogenous CAV1 (Fig. S1A). Cells were stably transfected with an empty plasmid (mock) or one containing an inducible insert that mildly increased CAV1 expression (approximately two-fold) [29] (Fig. S1B–D). As described [7], brief exposure (4 h) to the ER stressor tunicamycin increased mitochondria–ER proximity in mock cells, evaluated as colocalization using confocal microscopy. This adaptive increase, however, was abrogated by increased CAV1 expression (Fig. 1a, b), indicating that CAV1 interferes with the remodelling of ER–mitochondria contacts. To assess the functional consequences of this inhibition, we evaluated ER-to-mitochondria Ca^2+^ transfer by measuring mitochondrial Ca^2+^ increases elicited by histamine, an inducer of Ca^2+^ release from IP_3_-sensitive ER stores. In agreement with our colocalization data, early ER stress also increased Ca^2+^ transfer and this response was blunted by increased CAV1 expression (Fig. 1c, d). Importantly, changes in Ca^2+^ transfer were not due to alterations in the magnitude of Ca^2+^ release from the ER, as cells in all conditions displayed similar Ca^2+^ levels in response to histamine (Fig. 1e). As we previously reported [7], higher ER–mitochondria Ca^2+^ exchange during early ER stress resulted in higher mitochondrial respiration in mock cells. In contrast, increased CAV1 expression reduced the overall rates of mitochondrial respiration (Fig. 1f). Together, these data indicate that CAV1 prevents the mitochondrial response to early ER stress by modulating both mitochondrial bioenergetics and ER–mitochondria communication.

**Fig. 2:**
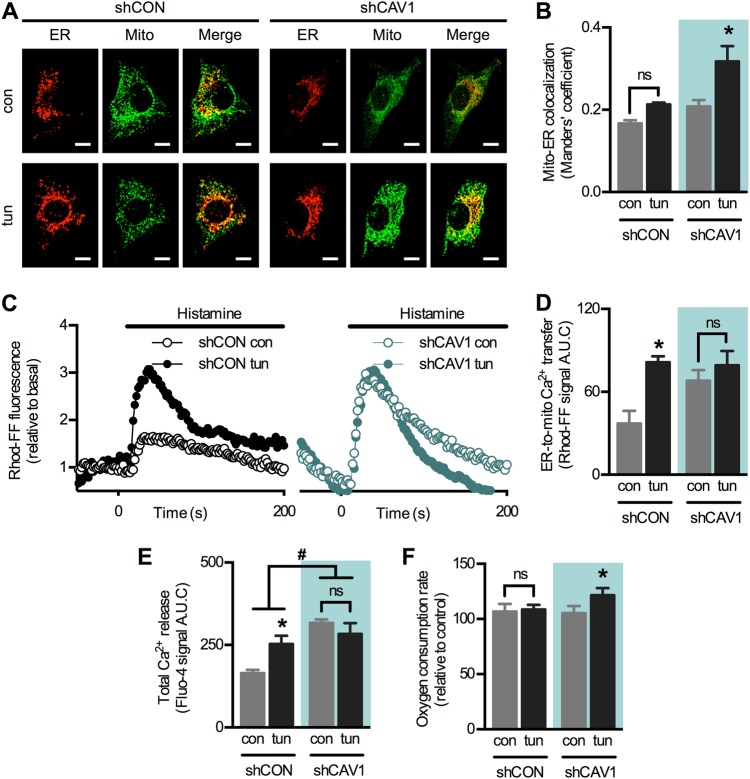
Caveolin-1 silencing enhances ER–mitochondria communication during the early phase of ER stress in MDA-MB-231 cells. **a** MDA-MB-231 shCON and shCAV1 cells in control condition (con) or under early ER stress with tunicamycin (tun) were processed for live-cell imaging. The ER was stained with ER-Tracker Red and mitochondria were stained with MitoTracker Green and then imaged using confocal microscopy. **b** Mitochondria–ER colocalization was quantified as Manders’ coefficients of images obtained in **a** (*n* = 3). **c** For experimental groups as in **a**, Ca^2+^ release from ER stores was induced with histamine, while mitochondrial Ca^2+^ levels were imaged with Rhod-FF using fluorescence microscopy. **d** Quantification of the area under the curve (A.U.C.) of graphs obtained in **c** (*n* = 3). **e** For cells treated as in **c**, Ca^2+^ release from ER stores was imaged with Fluo-4 using fluorescence microscopy and the resulting A.U.C. was quantified (*n* = 3). **f** For experimental groups as in **a**, mitochondrial respiration rates were measured using a Clark electrode (*n* = 3). For each independent imaging experiment, 5–15 cells were analysed. Scale bars: 10 µm. Results are shown as mean ± s.e.m. *P < 0.05 compared with respective con condition. ^#^*P* < 0.05 overall comparison between shCON and shCAV1 cells. ns non-significant

To validate our results, we resorted to another model using the opposite approach. MDA-MB-231 breast cancer cells, which express high levels of endogenous CAV1 (Fig. S1A), were stably transduced with either control (shCON) or CAV1-directed short hairpin RNA (shRNA) (shCAV1) as previously described [30] (Fig. S1E–G). In agreement with our previous study [8], early ER stress increased mitochondria–ER proximity in shCON cells; however, the magnitude of this increase was insignificant compared to shCAV1 cells (Fig. 2a, b). These data support the notion that CAV1 interferes with the formation of mitochondria–ER contact sites. Of note, basal mitochondria–ER colocalization was slightly higher in shCAV1 compared with shCON cells (Fig. 2b), suggesting that CAV1 silencing per se affects organelle communication. Likewise, lower levels of basal ER–mitochondria Ca^2+^ transfer were observed in shCON cells compared with shCAV1 cells (Fig. 2c, d). Rather surprisingly, tunicamycin increased Ca^2+^ transfer only in shCON but not in shCAV1 cells. Here, however, we also observed changes in total Ca^2+^ release from ER stores (Fig. 2e), and thus these findings may not reflect exclusively the efficiency of ER–mitochondria communication. In support of our hypothesis, ER stress increased mitochondrial respiration rate only in shCAV1 but not in shCON cells (Fig. 2f). These results suggest that CAV1 expression reduces mitochondria–ER proximity and metabolic responses to early ER stress. Given that CAV1 silencing in MDA-MB-231 cells altered baseline Ca^2+^ homeostasis (Fig. 2e), we considered HeLa cells a more suitable model to further explore the impact of CAV1 on ER–mitochondria communication.

**Fig. 3:**
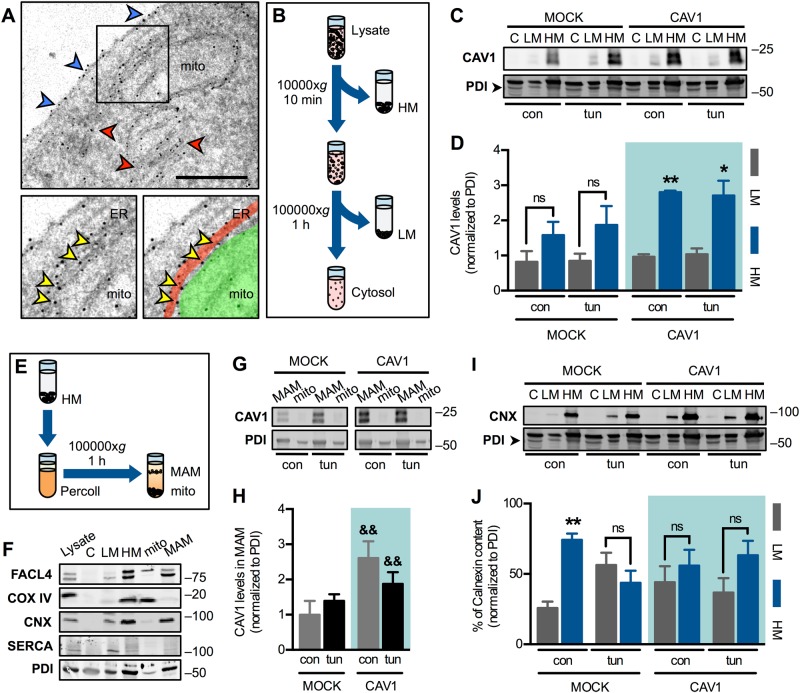
Caveolin-1 accumulates in mitochondria-associated ER membranes, precluding their remodelling upon early-phase ER stress. **a** Wild-type HeLa cells were labelled by immunogold staining with antibodies against CAV1. Blue arrowheads indicate CAV1 signals at the plasma membrane; red arrowheads indicate CAV1 signals at ER cisternae; yellow arrowheads indicate CAV1 at the ER–mitochondria interface. In the lower right panel, the ER is highlighted in red and mitochondria (mito) in green. Scale bar: 1 µm. **b** Cells lysates were fractionated by differential sedimentation. HM heavy membranes (crude mitochondria), LM light membranes (microsomes). **c** HeLa mock and CAV1 cells in control condition (con) or under early ER stress with tunicamycin (tun) were processed and fractionated as in **b**. CAV1 levels were analysed by western blotting using PDI as a loading control. **d** Quantification of membrane-bound CAV1 analysed in **c** (*n* = 3). **e** HM fraction obtained as in (**b**) was separated into MAM and pure mitochondria (mito) fractions by Percoll density gradient centrifugation. **f** Markers of cell fractionation procedures shown in **b** and **e**: FACL4 short isoform (mito), long isoform (MAM); COX IV (mito), Calnexin (CNX) (LM and MAM), SERCA2b (LM) and PDI (C, LM and HM). **g** Experimental groups as in **c** were processed and fractionated as in **e**. CAV1 levels were analysed by western blotting using PDI as a loading control. **h** Quantification of CAV1 in MAM analysed in **g** (*n* = 3). **i** Experimental groups as in **c** were processed and fractionated as in (**b**). CNX levels were analysed by western blotting using PDI as a loading control. **j** Quantification of membrane-bound CNX distribution analysed in **i** (*n* = 3). Results are shown as mean ± s.e.m. *P < 0.05 and **P < 0.01 compared with respective LM fraction. ^&&^*P* < 0.01 compared with respective mock condition. ns non-significant

Immunogold labelling of wild-type HeLa identified CAV1 at the plasma membrane, ER cisternae and also at the ER–mitochondria interface (Fig. 3a & S2). Accordingly, subcellular fractionation (Fig. 3b) of mock and CAV1-transfected HeLa cells revealed that CAV1 is present predominantly in heavy membranes (fraction containing mitochondria and MAM), and to a lesser extent in light membranes (microsomal fraction). Increased CAV1 expression resulted in its accumulation in heavy membranes, and ER stress did not affect this distribution (Fig. 3c, d). To gain further insight, we purified MAM and mitochondria from the heavy membranes fraction, as described [26] (Fig. 3e). As markers, we used FACL4 (long isoform enriched in mitochondria, short isoform enriched in MAM), COX IV (inner mitochondrial membrane protein), Calnexin (ER membrane protein enriched in MAM), SERCA2b (ER membrane protein) and PDI (soluble protein present in both mitochondria and ER and, to a lesser extent, in the cytosol) (Fig. 3f). In agreement with others [16], we observed the presence of CAV1 mainly in MAM rather than within the mitochondria fraction, indicating that the intracellular CAV1 pool is concentrated on the ER side of ER–mitochondria contacts (Fig. 3g, h). Taken together, our findings show that CAV1 expression levels, but not ER stress, determines the extent to which CAV1 is present in MAM. Consistent with our previous report [13], mock cells showed a characteristic Calnexin enrichment in MAM under resting condition, while early ER stress induced its translocation to light membranes as a compensatory response (Fig. 3i, j). In contrast, CAV1-transfected cells lacked the Calnexin enrichment in MAM, and this distribution did not change during early ER stress. These data suggest that CAV1 changes the properties of the ER–mitochondria interface and abolishes its ability to undergo remodelling upon ER stress.

### PKA mediates the remodelling of ER–mitochondria communication during early ER stress

**Fig. 4:**
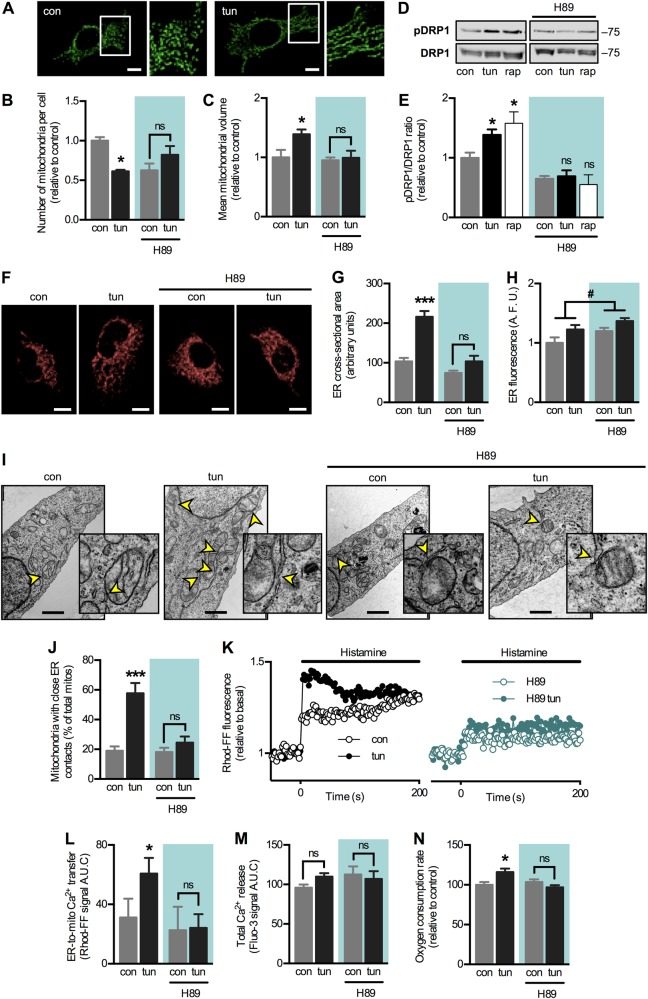
Early-phase ER stress induces PKA activation, which is required for organelle remodelling and to enhance ER–mitochondria communication. **a** Wild-type HeLa cells in control condition (con) or under early ER stress with tunicamycin (tun) were processed for live-cell imaging. Mitochondria were stained with MitoTracker Green and then imaged using confocal microscopy. **b** Quantification of the number of mitochondria per cell of images obtained in **a** in the absence or presence of the PKA inhibitor H89 (*n* = 3). **c** Quantification of the mean mitochondrial volume in cells treated and imaged as in (**b**) (*n* = 3). **d** Experimental groups as in (**b**) were analysed by western blotting. DRP1 Ser637 phosphorylation was normalized by total DRP1. Rapamycin treatment was used as a positive control. **e** Quantification of the DRP1 phosphorylation analysed in (**d**) (*n* = 3). **f** Experimental groups as in (**b**) were stained with ER-Tracker Red and then imaged using confocal microscopy. **g** Quantification of the cross-sectional area of the ER in images shown in (**f**) (*n* = 3). **h** Quantification of total fluorescence of the ER in images shown in **f** (*n* = 3). **i** Wild-type HeLa cells in control condition (con) or under early ER stress with tunicamycin (tun) in the absence or presence of H89 were imaged by electron microscopy. Arrowheads indicate ER–mitochondria contact sites. Scale bars: 1 µm. **j** Quantification of mitochondria in apposition to ER cisternae per cell from images as in **i**. Approximately 50 mitochondria were analysed for each independent sample. (**k**) For experimental groups as in **i**, Ca^2+^ release from ER stores was induced with histamine, while mitochondrial Ca^2+^ levels were imaged with Rhod-FF using fluorescence microscopy. **l** Quantification of the area under the curve (A.U.C.) of graphs obtained in **k** (*n* = 3). **m** For cells treated as in **l**, Ca^2+^ release from ER stores was imaged with Fluo-3 using fluorescence microscopy and the resulting A.U.C. was quantified (*n* = 3). **n** For experimental groups as in **i**, mitochondrial respiration rates were measured using a Clark electrode (*n* = 3). For each independent fluorescence microscopy experiment, 5–15 cells were analysed. Scale bars: 10 µm. Results are shown as mean ± s.e.m. *P < 0.05 and ***P < 0.001 compared with respective con condition. ^#^*P* < 0.05 overall comparison between H89-treated and untreated cells. ns non-significant

Next, we tested whether early ER stress-induced organelle remodelling depends on PKA signalling, as this kinase reportedly has pro-survival effects during stress [19, 23, 24]. As expected, we observed mitochondrial elongation in wild-type HeLa cells during early ER stress, as determined via three-dimensional (3D) reconstruction using confocal microscopy (Fig. 4a). Accordingly, we observed a decrease in the number of mitochondria per cell, concomitant with an increase in the mean mitochondrial volume (Fig. 4b, c), indicative of mitochondrial fusion. The PKA inhibitor H89 prevented both of these changes. Moreover, early ER stress induced DRP1 phosphorylation at Ser637, which, too, was abolished by H89 (Fig. 4d, e). As a positive control [19], we used the mTORC1 inhibitor rapamycin, with similar results. In addition to regulating mitochondrial dynamics, PKA and DRP1 have been associated with the regulation of ER morphology [25, 31, 32]. Given that ER stress induces expansion of the ER to cope with increased protein load [33], we tested whether PKA inhibition also alters ER morphology. Tunicamycin treatment led to expansion of the ER cross-sectional area, assessed by confocal microscopy (Fig. 4f–h). PKA inhibition with H89 prevented this increase, indicating that PKA activation during early ER stress not only regulates mitochondrial morphology but also ER dynamics.

Then we assessed whether PKA activation increases ER–mitochondria communication as part of its pro-survival actions. Electron microscopy showed that PKA inhibition with H89 prevented the increase in contacts in response to ER stress but did not affect the number of contacts in the basal state (Fig. 4i, j). To test MAM functionality, we evaluated ER–mitochondria Ca^2+^ transfer. As expected, PKA inhibition abrogated the increase in Ca^2+^ transfer induced by early ER stress (Fig. 4k, l) without affecting total Ca^2+^ release (Fig. 4m). Also, PKA inhibition prevented the increase in mitochondrial respiration arising from increased organelle communication (Fig. 4n). To discard the possibility that results obtained with H89 were due to potential off-target effects, we used a derivative from the endogenous-specific PKA inhibitor (PKI), with similar results. PKI abolished ER stress-induced increases both in organelle apposition and Ca^2+^ transfer (Fig. S3A–C). Moreover, PKI abrogated the increase in mitochondrial respiration due to ER stress. However, higher baseline values were also observed, indicating that this inhibitor per se alters mitochondrial bioenergetics (Fig. S3D). Given our previous results showing that the regulatory subunit RIIa determines PKA signalling in organelles [26], we silenced this subunit using siRNA (Fig. S3E). Similar to PKI, PKA RIIa silencing not only prevented the increase in mitochondrial respiration but also led to an apparent increase in mitochondrial respiration per se (Fig. S3F), possibly as a compensatory response by other PKA isoforms.

To further confirm that PKA was responsible for increased ER–mitochondria coupling during early ER stress, we used the adenylate cyclase (AC) activator forskolin to increase cAMP production and activate PKA. As expected, forskolin recapitulated the increase in mitochondrial respiration and a similar effect was observed with the DRP1 inhibitor Mdivi-1, which mimics the effect of PKA-mediated phosphorylation (Fig. S3G). Reportedly, AMPK is also activated during ER stress and phosphorylates DRP1 at Ser637 in β-pancreatic cells [25]. To determine whether AMPK contributes to our observations, we assessed the effects of both AMPK activation and inhibition. Indeed, the AMPK activator AICAR increased mitochondrial bioenergetics in HeLa cells; however, no further increases were observed in response to ER stress (Fig. S3H). Alternatively, the AMPK inhibitor compound C failed to prevent the increase in respiration induced by tunicamycin (Fig. S3I), ruling out the possibility that AMPK mediates the mitochondrial response to ER stress. In summary, these results show that PKA activity is required to increase ER–mitochondria contacts, as well as for ER expansion and mitochondrial elongation during the early phase of ER stress.

### CAV1 expression interferes with PKA-DRP1-mediated ER–mitochondria remodelling

**Fig. 5:**
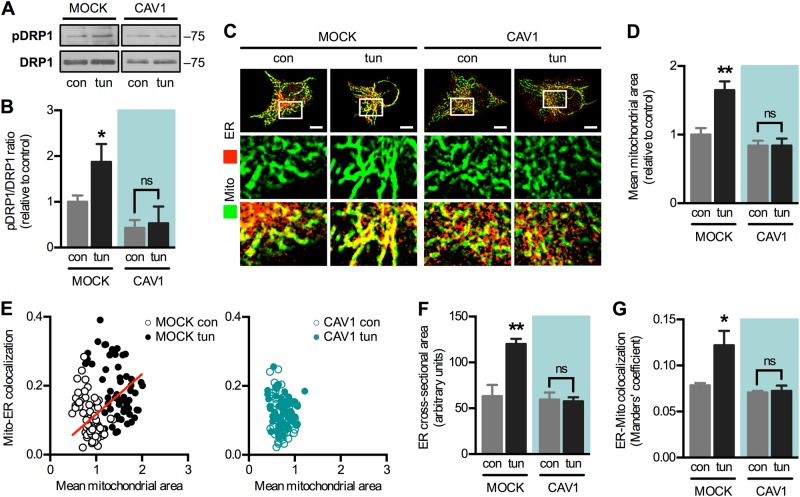
Caveolin-1 expression interferes with PKA-DRP1-associated organelle remodelling. **a** Mock and CAV1 cells in control condition (con) or under early ER stress with tunicamycin (tun) were analysed by western blotting. DRP1 Ser637 phosphorylation was normalized to total DRP1. **b** Quantification of the DRP1 phosphorylation analysed in **a** (*n* = 3). **c** Experimental groups as in **a** were processed for live-cell imaging. The ER was stained with ER-Tracker Red and mitochondria were stained with MitoTracker green and then imaged using confocal microscopy. **d** Mitochondrial mean area was quantified in images shown in (**c**) (*n* = 3). **e** Pearson’s correlation between mean mitochondrial size and ER–mitochondria proximity per cell was quantified in images shown in **c**. Cells were grouped according to CAV1 expression (mock and CAV1-transfected). Approximately 120 cells were analysed for each group. Only the mock group showed a significant correlation (*r* = 0.1926, *P* < 0.05). **f** Quantification of the cross-sectional area of the ER in images shown in **c** (*n* = 3). **g** ER–Mitochondria colocalization was quantified as Manders’ coefficients in images shown in **c** (*n* = 3). For each independent imaging experiment, 5–15 cells were analysed. Scale bars: 10 µm. Results are shown as mean ± s.e.m. *P < 0.05 and **P < 0.01 compared with respective con condition. ns non-significant

Given their opposing roles, we evaluated whether CAV1 interferes with the ability of PKA to modulate MAM. Indeed, increased CAV1 expression precluded both DRP1 phosphorylation (Fig. 5a, b) and mitochondrial elongation (Fig. 5c, d) induced during early ER stress. Interestingly, cells with elongated mitochondria also displayed higher extents of mitochondria–ER colocalization (Fig. 5c), suggesting that both processes are co-regulated. Accordingly, we detected a significant correlation between mitochondrial size and mitochondria–ER proximity in mock cells, which was abolished by CAV1 expression (Fig. 5e). Furthermore, ER expansion was also abrogated in CAV1 cells, indicating that the various aspects of PKA-driven organelle remodelling were compromised (Fig. 5f). Importantly, the increase in mitochondria–ER colocalization (Fig. 1b) was not simply the result of increased ER size, as the ER–mitochondria colocalization, which is normalized to total ER area, also increased in mock cells during ER stress but remained unaltered in CAV1 cells (Fig. 5g).

**Fig. 6:**
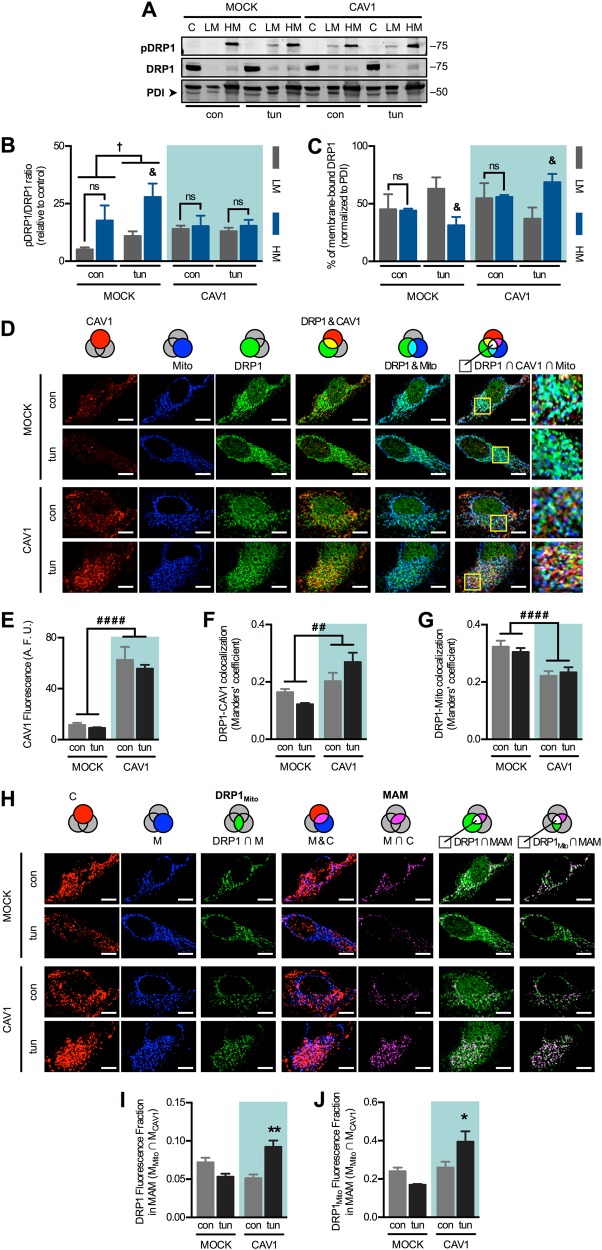
Caveolin-1 expression precludes DRP1 phosphorylation and redistribution during the early phase of ER stress. **a** Mock and CAV1 cells in control condition (con) or under early ER stress with tunicamycin (tun) were fractionated and processed for detection of phospho-DRP1 and DRP1 by western blotting using PDI as a loading control. C cytosol, LM light membranes (microsomes), HM heavy membranes (crude mitochondria). **b** Quantification of DRP1 phosphorylation analysed in (**a**) (*n* = 3). **c** Quantification of membrane-bound DRP1 distribution analysed in **a** (*n* = 3). **d** Experimental groups as in (**a**) were processed for immunofluorescence analysis. CAV1, mtHSP70 (mitochondrial marker, Mito) and DRP1 were stained with respective antibodies and then imaged using confocal microscopy. Merge between DRP1 and CAV1 images shows colocalized areas in yellow. Merge between DRP1 and Mito images shows colocalized areas in cyan. In merge images of Mito, DRP1 and CAV1, triple colocalization areas appear as white pixels. **e** CAV1 fluorescence per cell was quantified from images obtained in **d** (*n* = 3). **f** DRP1-CAV1 colocalization was quantified as Manders’ coefficients of images obtained in (**d**) (*n* = 3). **g** DRP1-Mito colocalization was quantified as Manders’ coefficients of images obtained in (**d**) (*n* = 3). **h** From images obtained in **d**, thresholded CAV1 (C) and Mito (M) images were generated. The intersection between M and DRP1 corresponds to mitochondrial DRP1 (DRP1_mito_). Merge between M and C images shows colocalized areas in magenta. The intersection between M and C corresponds to MAM. Merge between MAM and DRP1 or DRP1_mito_ images shows colocalized pixels in white, which correspond to triple colocalization areas. **i** The fraction of DRP1 fluorescence that colocalizes with MAM was quantified from images obtained in **h** (*n* = 3). **j** The fraction of DRP1_mito_ fluorescence that colocalizes with MAM was quantified from images obtained in **h** (*n* = 3). For each independent imaging experiment, 5–15 cells were analysed. Scale bars: 10 µm. Results are shown as mean ± s.e.m. ^&^*P* < 0.05 compared with respective LM fraction. ^†^*P* < 0.05 overall comparison between conditions. *P < 0.05 and **P < 0.05 compared with respective con condition. ^##^*P* < 0.01 and ^####^*P* < 0.0001 overall comparison between conditions. ns non-significant

Because CAV1-induced alterations were linked to PKA effects, we analysed the subcellular distribution and phosphorylation of its substrate, DRP1. This protein was predominantly cytosolic under all conditions and only present in low amounts in light and heavy membrane fractions (Fig. 6a). In response to ER stress, overall DRP1 phosphorylation increased but was particularly higher in the heavy membrane fractions (Fig. 6b). In contrast, DRP1 phosphorylation remained unchanged upon induction of ER stress in CAV1 cells. In terms of total distribution, mock cells showed DRP1 translocation towards light membranes during ER stress (Fig. 6c), which may be associated with the changes in ER and mitochondrial morphology. In CAV1 cells, on the contrary, DRP1 was enriched in heavy membranes, which likely precludes the said changes. To confirm these observations, we performed triple colocalization analysis of immunofluorescence of DRP1, CAV1 and mitochondria (probed with mitochondrial heat-shock protein-70 (mtHSP70)) (Fig. 6d). As expected, mock cells displayed lower CAV1 fluorescence levels and hence lower DRP1-CAV1 colocalization (Fig. 6e, f). Similar to our fractionation analysis, we observed a tendency for DRP1-CAV1 colocalization to decrease in mock cells upon ER stress, while CAV1 expression led to the opposite response. Overall DRP1-mitochondria colocalization was higher in mock cells compared to CAV1 cells (Fig. 6g). We used the thresholded images of CAV1 and mitochondria to obtain their intersection, which represents a way of visualizing MAM, while the intersection between DRP1 and thresholded mitochondria corresponds to mitochondria-bound DRP1 (Fig. 6h). In agreement with our fractionation analysis, both total DRP1 as well as mitochondrial DRP1 fluorescence in MAM slightly decreased in response to ER stress in mock cells, while, on the contrary, both values significantly increased in cells with augmented CAV1 expression (Fig. 6i, j). Altogether, these results indicate that, during early ER stress, CAV1 antagonizes DRP1 phosphorylation and redistribution between ER and mitochondria. As a potential mechanism linking CAV1 expression to decreased PKA-mediated phosphorylation, we analysed AC5/6 localization, considering that it is a membrane-bound activator of PKA that associates with and is inhibited by CAV1 [34–36]. Triple colocalization experiments showed that the total amount of AC5/6 in mitochondria does not change with ER stress in either mock or CAV1 cells (Fig. S4A–C). Rather, early ER stress induces mitochondria-associated AC5/6 redistribution from CAV1-containing domains to CAV1-free mitochondria in mock cells (Fig. S4D–E). This change might be associated with local PKA activation and higher DRP1 phosphorylation. Alternatively, in CAV1 cells, no such AC5/6 redistribution was observed, which might be associated with decreased cAMP production and reduced adaptation to ER stress.

**Fig 7:**
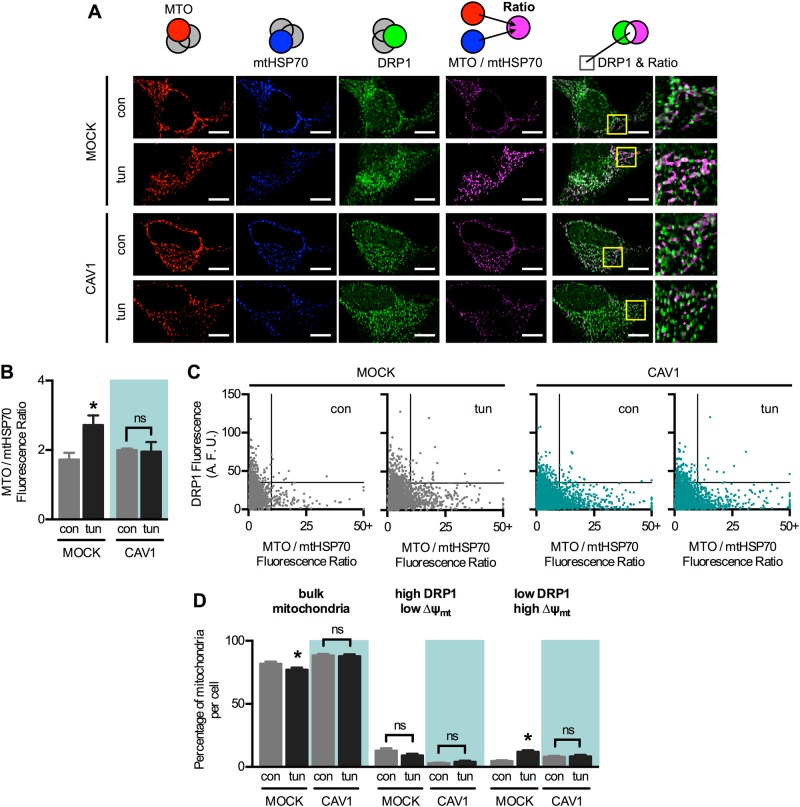
DRP1 redistribution during the early phase of ER stress correlates with increased mitochondrial bioenergetics. **a** Mock and CAV1 cells in control condition (con) or under early ER stress with tunicamycin (tun) were processed for immunofluorescence analysis. The mitochondrial potential was stained with MitoTracker Orange (MTO) and mtHSP70 (mitochondrial marker) and DRP1 were stained with the respective antibodies and then imaged using confocal microscopy. The ratio between MTO and mtHSP70 is shown in magenta. Merge between DRP1 and ratio images shows colocalized areas in white. **b** MTO/mtHSP70 fluorescence ratio per cell was quantified from images obtained in **a** (*n* = 3). **c** Dot plots of DRP1 fluorescence and MTO/mtHSP70 ratio of each individual mitochondria, obtained from images shown in (**a**) (*n* = 3). Between 2000 and 6000 individual mitochondria per condition were analysed. **d** Percentage of mitochondria per cell corresponding to the quadrants defined in **a**. Between 25 and 45 cells were pooled for this analysis. ∆ψ_mt_: MTO/mtHSP70 ratio. Bulk mitochondria: low DRP1 and low ∆ψ_mt_. For each independent imaging experiment, 5–15 cells were analysed. Scale bars: 10 µm. Results are shown as mean ± s.e.m. *P < 0.05 compared with respective con condition. ns non-significant

To address the relevance of DRP1 presence in mitochondria, we performed yet another immunofluorescence analysis, this time between mtHSP70, DRP1 and MitoTracker orange (MTO), a mitochondrial transmembrane potential (∆ψ_mt_)-sensitive probe (Fig. 7a). The ratio between MTO and mtHSP70 is a measure of ∆ψ_mt_, which increases in mock cells upon ER stress, as previously reported [7, 8] (Fig. 7b). CAV1 expression, again, precluded that adaptive change. Then, we analysed the fluorescence levels in individual mitochondria of all imaged cells (Fig. 7c). In control mock cells, the bulk of mitochondria displayed relatively low ∆ψ_mt_ and low DRP1 fluorescence. Mitochondria with higher DRP1 displayed low ∆ψ_mt_, while mitochondria with higher ∆ψ_mt_ showed low DRP1 levels. Upon early ER stress, the mitochondrial population with high ∆ψ_mt_ and low DRP1 increased, at the expense of the bulk mitochondria population (Fig. 7d). In CAV1 cells, however, there was no change in mitochondrial populations in response to ER stress. These data indicate that DRP1 redistribution out of mitochondria is associated with enhanced mitochondrial function, which is abrogated by CAV1.

### Restoring ER–mitochondria apposition reverts CAV1-induced sensitivity to ER stress

**Fig 8:**
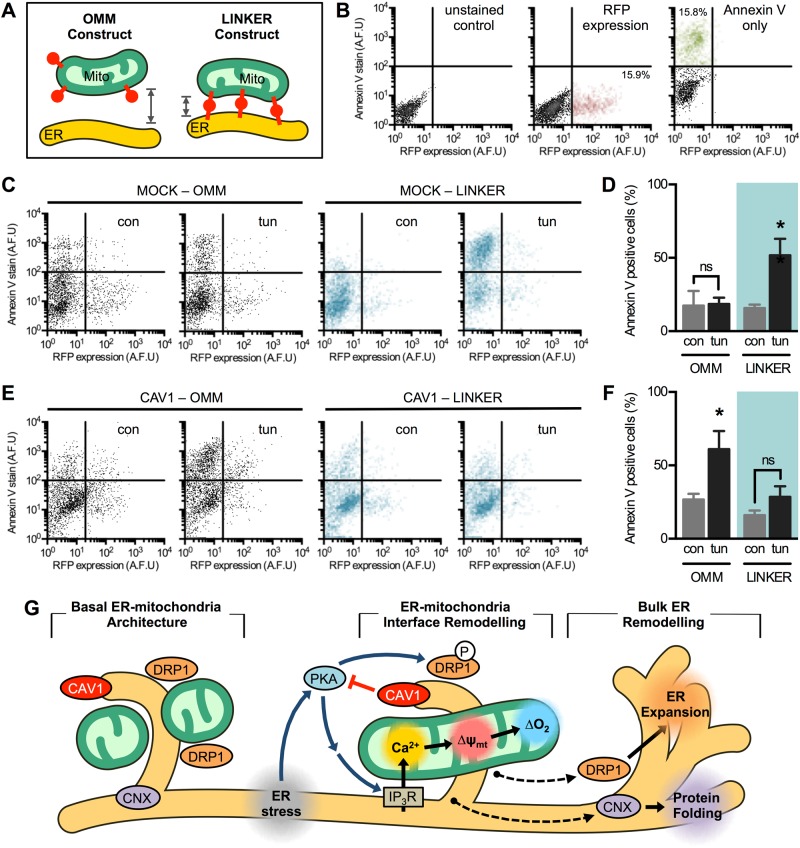
Artificial ER–mitochondria cross-linking protects cells against Caveolin-1-induced maladaptation to ER stress. **a** Cells were transfected with RFP targeted to the OMM or simultaneously to the OMM and the ER, the latter resulting in an artificial linker that enforces apposition between both organelles. **b** Representative dot plots of unstained HeLa cells or transfected with a RFP construct or stained for annexin V detected by flow cytometry. **c** Representative histograms of mock cells transfected with either OMM-RFP or OMM-RFP-ER constructs, then subjected to control condition (con) or to cytotoxic ER stress (tun). After 24 h, annexin V staining was measured in RFP-positive cells by flow cytometry. **d** Quantification of annexin V staining in RFP-positive cells obtained in **c**. **e** Representative histograms of CAV1 cells were treated and analysed as cells in **c**. **f** Quantification of annexin V staining in RFP-positive cells obtained in **e**. Results are shown as mean ± s.e.m. *P < 0.05 compared with respective con condition. ns non-significant. **g** CAV1 blocks PKA-mediated adaptive ER–mitochondria responses to ER stress. In basal conditions, CAV1, DRP1 and CNX are enriched in the ER–mitochondria junction. Early ER stress induces MAM remodelling, observed as CNX translocation out of MAM to the bulk ER. Early ER stress also triggers PKA activation, thereby leading to ER expansion and mitochondrial elongation via DRP1 phosphorylation, as well as increased ER to mitochondria Ca^2+^ transfer and enhanced mitochondrial metabolism. CAV1, on the other hand, alters MAM protein composition, as in the case of CNX by increasing its presence in the bulk ER and thus reducing MAM adaptability to ER stress. CAV1 expression also impairs PKA signalling, thus precluding the adaptive changes in both ER and mitochondria morphology and communication in response to ER stress. Therefore, CAV1 negatively regulates the remodelling of ER–mitochondria communication required for cells to adjust to ER stress

Given that CAV1 disrupts ER–mitochondria communication associated with adaptation to ER stress, we explored whether artificially inducing ER–mitochondria proximity using a recombinant linker [37] might suffice to revert CAV1-induced anomalies. Both mock and CAV1-overexpressing cells were transfected with the linker, which contains red fluorescent protein (RFP) targeted simultaneously to the outer mitochondrial membrane (OMM) and the ER (OMM-RFP-ER). As a control, OMM-targeted RFP was used (Fig. 8a). Then, cells were treated with a cytotoxic dose of tunicamycin. Given that our transfection rate was ~15%, we measured cell death as annexin V staining in RFP-expressing cells using flow cytometry (Fig. 8b). In mock cells, introduction of the linker OMM-RFP-ER increased ER stress-induced cell death as compared with the control OMM-RFP (Fig. 8c, d). This observation confirms the reported finding that excessive ER–mitochondria proximity triggers cell death via mitochondrial Ca^2+^ overload [37]. In contrast, CAV1 cells were more sensitive to ER stress, which was completely reverted by expression of the linker (Fig. 8e, f), indicating that restoring ER–mitochondria contacts recovers cells' resistance to ER stress. In agreement with these results, MDA-MB-231 shCON cells were more sensitive to ER stress-induced cell death compared with shCAV1 cells (Fig. S5A-D). Together, these data highlight CAV1 as a negative regulator of ER–mitochondria communication and remodelling, both key factors for cell adaptation to ER stress.

## Discussion

Here we report that increased CAV1 expression precludes the increase in ER–mitochondria contacts during early ER stress, while CAV1 silencing has the opposite effect. These results partially agree with Sala-Vila et al. showing that MAM from CAV1-knockout mice have elevated cholesterol levels [16], which exacerbate the function of the respiratory chain [18]. Our results, however, contrast with respect to organelle proximity, as we find that CAV1 reduces ER–mitochondria communication, while they report that CAV1 is required for MAM formation [16]. These apparent discrepancies might arise because different models were studied. While we used an inducible CAV1 expression system, they studied a genetic knockout model, where the observed responses might be due to compensation to CAV1 deletion [18]. Moreover, we studied cancer cell lines, while they focussed on liver cells. As a central organ for cholesterol homeostasis, it is not surprising that the liver may respond distinctly to changes in CAV1 expression compared with other cells/tissues. Indeed, ER–mitochondria contacts reportedly decrease during pathogenic processes in the heart [9], vasculature [10] and brain [11], while they increase in the liver [12].

In the context of cancer, our findings agree with the proposed role for CAV1 in “metabolic synergy” of solid tumours. In stromal cells, loss of CAV1 expression promotes mitochondrial metabolism, thereby increasing oxidative stress, genomic instability and cell transformation, which is consistent with the notion that CAV1 functions as a tumour suppressor [29, 38, 39]. Metabolite exchange between normal stromal cells and mitochondria-dependent transformed cells fuels tumour growth, a process known as the Warburg effect [40]. In contrast, CAV1 re-expression in advanced tumours has been shown to promote metastasis [39, 41], although this effect seems to be rather a consequence of extra-mitochondrial CAV1 signalling [42]. Work on H-Ras-transformed cells demonstrated that CAV1 also acts as a tumour suppressor by increasing intracellular Ca^2+^ levels to trigger cell death [43]. Here we show that CAV1 reduces ER–mitochondria Ca^2+^ transfer, which may hinder the mitochondrial Ca^2+^ buffering capacity, thus contributing to Ca^2+^-mediated cell death [4]. Indeed, increased CAV1 expression leads to higher sensitivity to cell death upon ER stress, and artificially increasing ER–mitochondria contacts in these cells restores viability. Conversely, mock cells adapt to ER stress by enhancing ER–mitochondrial coupling; however, further increases with the ER–mitochondria linker decreases cell survival, via mitochondrial Ca^2+^ overload [37, 44]. These observations highlight the complex nature of the ER–mitochondria interface, as either increasing or decreasing contacts can be deleterious. However, one of the limitations of our study is that we were unable to determine whether the observed effects on mitochondrial metabolism were due to CAV1 localizing specifically at MAM or elsewhere in the cell. Because of that, extra-mitochondrial CAV1 effects cannot be discarded.

We show that, during early ER stress, DRP1 translocates to ER membranes, while PKA-phosphorylated DRP1 accumulates at MAM. These events are associated with mitochondrial elongation, ER expansion and increased ER–mitochondria contacts. Taken together, these results indicate that PKA is a common stress mediator that synchronizes ER and mitochondrial responses via DRP1. The observation that phospho-DRP1 reportedly has reduced mitochondrial-constriction activity, yet is still detected at MAM, suggests two non-exclusive possibilities: (a) phospho-DRP1 is basally assembled at MAM, ready to act upon dephosphorylation, and/or (b) phospho-DRP1 participates in ER–mitochondria contact formation; nonetheless, the mechanism behind this remains to be determined.

In summary (Fig. 8g), we provide evidence that CAV1 is present at MAM and inhibits the remodelling of ER–mitochondria contacts that is mediated by the PKA-DRP1 signalling axis. Early ER stress induces PKA-dependent DRP1 phosphorylation, thereby promoting mitochondrial elongation. DRP1 also redistributes to microsomal membranes during ER stress, probably to participate in ER expansion. Furthermore, PKA activity is required for ER–mitochondria contact formation and Ca^2+^ transfer, ultimately enhancing mitochondrial bioenergetics. Modulation of ER–mitochondria proximity greatly influences cell death and survival during ER stress, with opposite outcomes depending on the CAV1 expression levels. In summary, these observations position the CAV1–PKA–DRP1 axis as a key regulator of organelle communication during ER stress.

## Materials and Methods

### Reagents

Chemicals for general-purpose solutions were from Merck Millipore (Burlington, MA, USA). All other reagents were from Thermo Fisher Scientific (Waltham, MA, USA), unless otherwise stated.

### Cell culture

Wild-type cell lines were obtained from American Type Culture Collection (ATCC), Manassas, VA, USA. HeLa and MDA-MB-231 cells were maintained in Dulbecco’s modified Eagle’s medium (DMEM; Sigma-Aldrich, St Louis, MO, USA) and DMEM-F12, respectively. Both media were supplemented with 10% foetal bovine serum and penicillin–streptomycin–amphotericin B antibiotics (Biological Industries, Beit HaEmek, Israel). Cells were cultured in a 5% CO_2_ atmosphere at 37 °C.

### CAV1 expression

HeLa cells were stably transfected with the vector pLacIOP plasmid alone (mock) or with a CAV1-encoding IPTG-inducible insert, as previously described [29]. We obtained two mock cell lines that, due to clonal differences, expressed lower CAV1 compared to parental (P) HeLa cells (Fig. 1a). We performed all experiments using clone #1 in which CAV1 levels were lower. Following transfection with CAV1-encoding vector, we obtained one clone, in which a mild increase in CAV1 levels was detected following induction compared to parental cells. Prior to all experimentation, CAV1 expression was induced using 1 mM IPTG (Sigma-Aldrich) for 24 h. MDA-MB-231 cells were stably transduced with the lentiviral vector pLKO.1 containing luciferase (shCON cells) or CAV1-directed shRNA (shCAV1 cells), as previously described [30].

Every 3 weeks, HeLa and MDA-MB-231 cells were selected for 7–10 days with either 500 µg/mL hygromycin or 2 µg/mL puromycin, respectively, to ensure plasmid maintenance.

### Transient transfection

Cells were seeded in 6-well dishes at 60% confluence and transfected using OptiMEM and Lipofectamine 2000, according to the manufacturer’s specifications. Cells were transfected with plasmids bearing either OMM-targeted RFP or RFP targeted to both the ER and the OMM [37]. Both plasmids were a kind donation from Dr. György Hajnóczky from Thomas Jefferson University, Philadelphia, PA, USA. In the case of control siRNA #SIC001 (Sigma-Aldrich) or siRNA against PKA RIIa #SIHK1812 (Sigma-Aldrich), cells were transfected using OptiMEM and Lipofectamine RNAiMAX according to the manufacturer’s specifications. Transfected cells were maintained for 24 h prior to further experimentation, to ensure adequate protein expression or silencing.

### Experimentation

To study the adaptive response to early ER stress, cells were treated with tunicamycin (Enzo Life Sciences, Farmingdale, NY, USA) at a non-lethal dose (0.5 µg/mL) for 4 h. To assess ER stress-induced cell death, HeLa cells were treated with a higher dose (10 µg/mL) for 24 h. In the case of MDA-MB-231 cells, cytotoxic ER stress was achieved with 0.5 µg/mL for 24 h. Other stimuli, all for 4 h, were as follows: rapamycin (Sigma-Aldrich) 100 nM, H89 (Calbiochem, La Jolla, CA, USA) 10 µM, forskolin (Sigma-Aldrich) 100 µM, Mdivi-1 (Sigma-Aldrich) 50 µM, AICAR (Sigma-Aldrich) 250 µM, compound C (Sigma-Aldrich) 100 nM, and PKI [14-22]-myr (Thermo Fisher Scientific) 10 µM. To induce Ca^2+^ release from ER stores, histamine (Sigma-Aldrich) was used at 100 µM.

### Total protein extracts

Cells were seeded in 60-mm dishes at 80% confluence and treated according to the experiment. Cells were lysed with a mild buffer (10 mM Tris-HCl pH 7.4; 5 mM EDTA; 50 mM NaCl; 0.5% v/v NP40) in the presence of protease and phosphatase inhibitor cocktails (Roche, Basel, Switzerland). Homogenates were centrifuged at 8000 × *g* for 10 min to eliminate cellular debris including nuclei. Protein concentrations were measured using the Bradford method according to the manufacturer’s instructions (Bio-Rad, Hercules, CA, USA). Protein extracts were denaturated with Laemmli buffer (62.5 mM Tris-Base pH 6.8; 8% glycerol; 2.3% sodium dodecyl sulfate (SDS); 0.005% bromophenol blue; 5% 2-mercaptoethanol) for 5 min at 100 °C, then stored at –20 °C.

### Western blot analysis of total protein extracts

Protein extracts were separated by SDS–polyacrylamide gel electrophoresis (10% gels) at room temperature at 100 mV and then transferred to 0.2-µm-pore nitrocellulose membranes (Macherey-Nagel, Düren, Germany) at 4 °C at 400 mA using a Mini-PROTEAN Tetra Cell and a PowerPac Basic, both from Bio-Rad. Membranes were blocked with 5% non-fat milk 0.05% Tween 20 TBS for 1 h at room temperature, then incubated with primary antibodies overnight at 4 °C. Antibody dilutions were: anti-CAV1 #610060 (BD Transduction Laboratories, San Jose, CA, USA) 1:3000; anti-ACTB #A5316 (Sigma-Aldrich) 1:5000; anti-pDRP1 #4867 (Cell Signaling Technology, Danvers, MA, USA) 1:500; anti-DRP1 #611113 (BD Transduction Laboratories) 1:1000; and anti-PKA RIIa, #MA3-517 (Thermo Fisher Scientific) 1:1000. After washing blots in 0.05% Tween TBS, blots were incubated for 2 h with anti-mouse or anti-rabbit peroxidase-conjugated secondary antibodies (Calbiochem) at dilution 1:5000. Protein bands were detected using EZ-ECL reagents (Biological Industries) and either scanned with a G-BOX (Syngene, Bangalore, India) or developed to X-ray films (Agfa-Gevaert, Mortsel, Belgium). ImageJ software (National Institute of Health, Rockville, MD, USA) was used for densitometric analysis.

### Immunofluorescence

Cells were seeded in 12-well plates with 0.17-mm coverslips at 30% confluence and treated as indicated in each experiment. For staining with MTO, the probe was added to the cells at 400 nM and incubated for 20 min prior to fixation. Cells were then fixed with 4% paraformaldehyde (Electron Microscopy Sciences, Hatfield, PA, USA), permeabilized with 0.1% Triton X-100 (Sigma-Aldrich) and blocked with 3% bovine serum albumin (BSA), all in PBS. Samples were incubated with primary antibodies in 3% BSA overnight at 4 °C. Antibody dilutions were: anti-CAV1 #610060 (BD Transduction Laboratories) 1:100; anti-DRP1 #611113 (BD Transduction Laboratories) 1:500; anti-mtHSP70 #PA548035 (Thermo Fisher Scientific) 1:50; and anti-AC5/6 #ab66037 (Abcam) 1:200. Following incubation for 2 h with anti-mouse, anti-rabbit or anti-goat Alexa-conjugated secondary antibodies, coverslips were mounted on glass slides using mounting medium (DAKO Corporation, Carpinteria, CA, USA) as described [45].

### Live-cell microscopy

Cells were seeded in 6-well plates with 0.17-mm coverslips at 30% confluence and treated as required in each experiment. Cells were incubated with Krebs medium (10 mM HEPES pH 7.4; 145 mM NaCl; 5 mM KCl; 2.6 mM CaCl_2_; 1 mM MgCl_2_; 5.6 mM glucose) containing the desired fluorescent probes for 30 min at 37 °C. For ER and mitochondrial network staining, ER-Tracker Red and MitoTracker Green were used. Rhod-FF-AM (5.5 µM) was used for mitochondrial Ca^2+^ imaging, while Fluo-3-AM (4.4 µM) or Fluo-4-AM (4.6 µM) were used for cytoplasmic Ca^2+^ imaging in HeLa and MDA-MB-231 cells, respectively, as previously described [8, 46].

### Image acquisition and processing

Fixed or live cells were imaged using a Zeiss LSM 5, Pascal Axiovert 200 confocal microscope (Carl Zeiss, Oberkochen, Germany), with a Plan-Apochromat ×63/1.4 Oil DIC objective and 488, 543 and 639 nm excitation lasers. In each independent experiment, 5–15 cells were evaluated and averaged [7]. For dynamic calcium measurements, images were acquired at 1 s intervals. Basal fluorescence was measured for 50 s, and then histamine-induced signals were imaged for 200 s. Data are expressed as fluorescence change relative to basal values ([*F* − *F*_0_]/*F*_0_). The area under the curve was quantified during the first 50 s post-stimulus. For static fluorescence measurements, images were deconvoluted, background-subtracted, thresholded and analysed using the ImageJ software. Colocalization analysis was performed within a single plane at the cell equator using the JACoP plugin. 3D object analysis was performed on cell reconstructions consisting of 10 *z*-planes using the 3D Object Counter plugin. ER cross-sectional area, mean mitochondrial area, mitochondrial DRP1 fluorescence and MTO/mtHSP70 fluorescence ratio per mitochondria were analysed within a single plane at the cell equator using the Analyze Particles function. For triple colocalization analysis, image intersections were obtained using the Image Calculator command of ImageJ (“AND” operator).

### Oxygraphy

Cells were seeded in 60-mm dishes at 80% confluence and treated according to the experiment. Cells were trypsinized and the resulting suspension was placed in a chamber with a Clark electrode (Strathkelvin Instruments, North Lanarkshire, Scotland), which measures oxygen consumption in living cells. After measuring basal respiration for 3 min, CCCP 200 nM was added to measure uncoupled respiration for another 3 min, as described [8, 47].

### Electron microscopy

Cells were seeded in 35-mm dishes at 30% confluence and treated according to the experiment. Following fixation with 2.5% glutaraldehyde, samples were embedded in 2% agarose, post-fixed in 1% osmium tetroxide, stained en-bloc with uranyl acetate and dehydrated using ethanol. Ultimately, samples were embedded in Epon 812 resin and cut using a Leica Ultracut UCT ultramicrotome (Leica Biosystems, Wetzlar, Germany). Imaging was performed using a FEI Tecnai G^2^ Spirit electron microscope as described [7, 8].

### Immunogold

Cells grown to ~80% confluence in 35-mm dishes were fixed with 3% paraformaldehyde 0.05%, glutaraldehyde and 0.5 M sucrose. After permeabilization with saponin, samples were blocked and incubated with anti-CAV1 antibody #610407 (BD Transduction Laboratories). After incubation with anti-mouse FluoronaNogold Alexa Fluor 594 secondary antibody (NanoProbes, Yaphank, NY, USA), samples were fixed again with 2% glutaraldehyde and 0.5 M sucrose. Enlargement of 1.4 nm Nanogold particles was achieved using GoldEnhance EM Plus #2114 (NanoProbes). Cells were then scraped from the dishes, centrifuged to make pellets and post-fixed in osmium tetroxide, followed by overnight incubation with 1.25% uranyl acetate. Following dehydration, pellets were embedded in EMbed 812 (Electron Microscopy Sciences), sectioned using an Ultracut E ultramicrotome (Reichert Technologies, Depew, NY, USA) and imaged using a JEM-2100 electron microscope (JEOL Ltd, Tokyo, Japan).

### Cell fractionation and analysis

Cells were seeded in fifteen 200-mm dishes at 80% confluence per condition and treated according to the experiment. Cells were gently scraped from the plate surface in homogenization buffer (10 mM HEPES pH 7.6; 1 mM EDTA; 250 mM Sucrose) containing protease and phosphatase inhibitor cocktails. Samples were homogenized using a bearing-ball homogenizer (Isobiotec, Heidelberg, Germany) and centrifuged at 600 × *g* for 10 min at 4 °C to discard debris and nuclei. Supernatants were then centrifuged at 10,000 × *g* for 10 min at 4 °C to pellet crude mitochondria. Resulting supernatants were centrifuged at 100,000 × *g* for 1 h at 4 °C to separate cytosol (supernatant) from microsomes (pellet). Crude mitochondria fractions were resuspended in 1 mL homogenization buffer, layered onto 7.9 mL 18% Percoll homogenization buffer and centrifuged at 95,000 x *g* for 30 min at 4 °C in a 90Ti rotor (Beckman Coulter, Brea, CA) to yield a lighter MAM and a heavier mitochondrial fraction [26]. Proteins from each fraction were precipitated overnight with acetone, dried, resuspended in Laemmli buffer for 5 min at 100 °C and then stored at –20 °C. Samples were analysed by western blotting as described for total protein extracts, with some differences. Antibody dilutions were: anti CAV1 #610059 (BD Transduction Laboratories) 1:1000; anti PDI #MA3019 (Thermo Fisher Scientific) 1:5000; CNX antiserum 1:1000; anti FACL4 #110007 (Abcam, Cambridge, UK) 1:2000; anti COX IV #4850 (Cell Signaling Technology) 1:1000; anti SERCA #MAB2636 (Merck Millipore) 1:1000; anti pDRP1 #4867 (Cell Signaling Technology) 1:5000; and anti DRP1 #56788 (Abcam) 1:1000. Following incubation for 2 h with anti-mouse, anti-rabbit or anti-goat Alexa-conjugated secondary antibodies at a 1:5000 dilution, protein bands were scanned with an Odyssey infrared imaging system (LICOR, Lincoln, NE, USA).

### Cell viability and survival assays

For these experiments, cells were seeded in 12-well plates and then subjected to experimental conditions. Loss of cell viability in HeLa cells, determined as phosphatidylserine exposure on the cell surface, was measured using a fluorescent annexin V-based kit (BD Biosciences, San Jose, CA, USA), following the manufacturer’s specifications. In the case of MDA-MB-231 cells, viability was assessed using 1 µg/mL propidium iodide (Sigma-Aldrich). Cell fluorescence was measured by flow cytometry (FACS Canto II, BD Biosciences).

### Statistical analysis

Results are mean ± s.e.m. of at least three independent experiments. Statistical significance was determined using a 95% confidence level (*P* < 0.05). For comparisons between three experimental groups (con/tun/rap, con/forsk/mdivi), one-way analysis of variance (ANOVA) was used followed by a Bonferroni post-test. For comparisons between two groups (con/tun) in combination with a stratifying factor (MOCK/CAV1, shCON/shCAV1, LM/HM, OMM/LINKER or presence/absence of inhibitor), two-way ANOVA was used followed by Holm–Sidak post-test. For correlation analysis, a two-tailed Pearson’s correlation coefficient was calculated.

## Electronic supplementary material

Supplementary figure 1

Supplementary figure 2

Supplementary figure 3

Supplementary figure 4

Supplementary figure 5

supplementary figure legends
