# Supplementary figures and images for: Caveolin-1 impairs PKA-DRP1-mediated remodelling of ER–mitochondria communication during the early phase of ER stress
Source: Cell Death Differ. 2018 Sep 12;26(7):1195–212. doi: 10.1038/s41418-018-0197-1 (PMC6748148; doi:10.1038/s41418-018-0197-1)

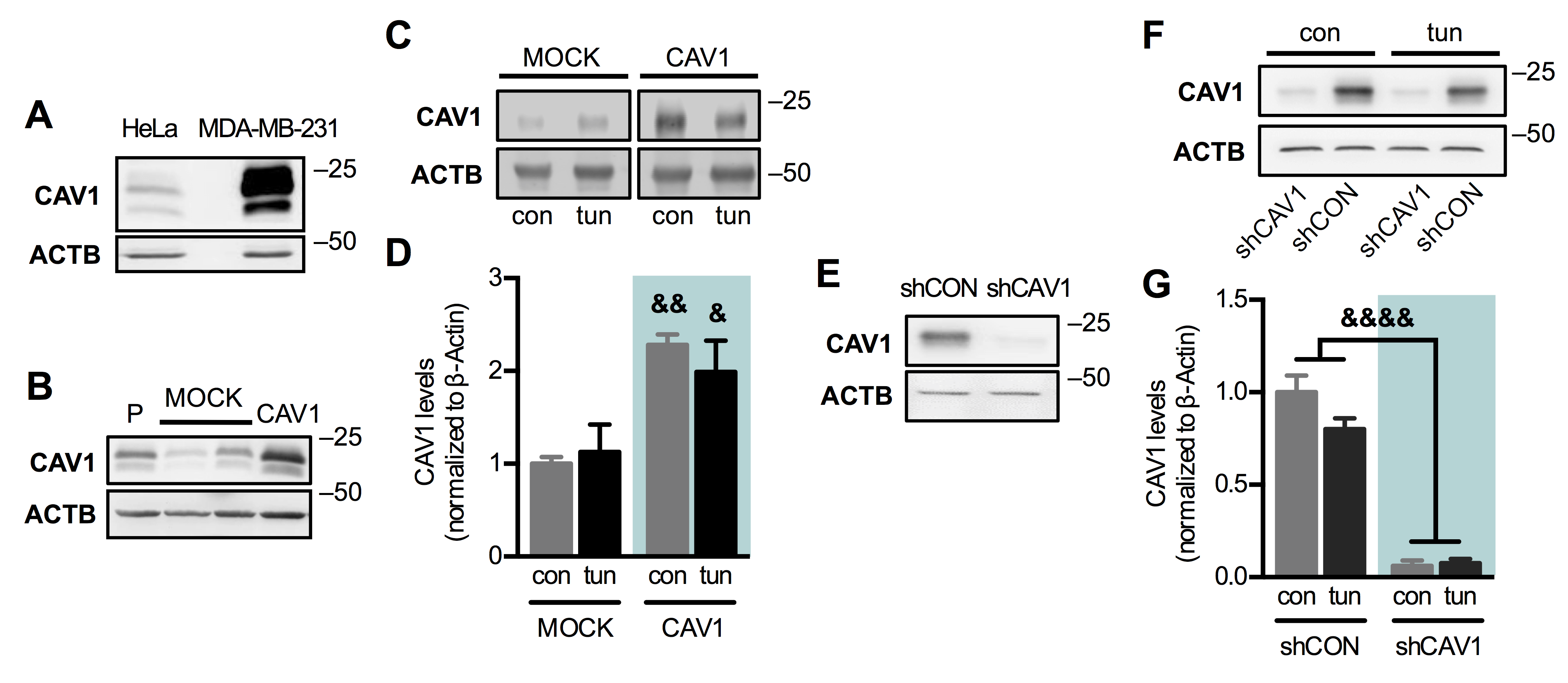

Supplement: Supplementary file 1 — Supplementary figure 1 [file 41418_2018_197_MOESM1_ESM.tif]

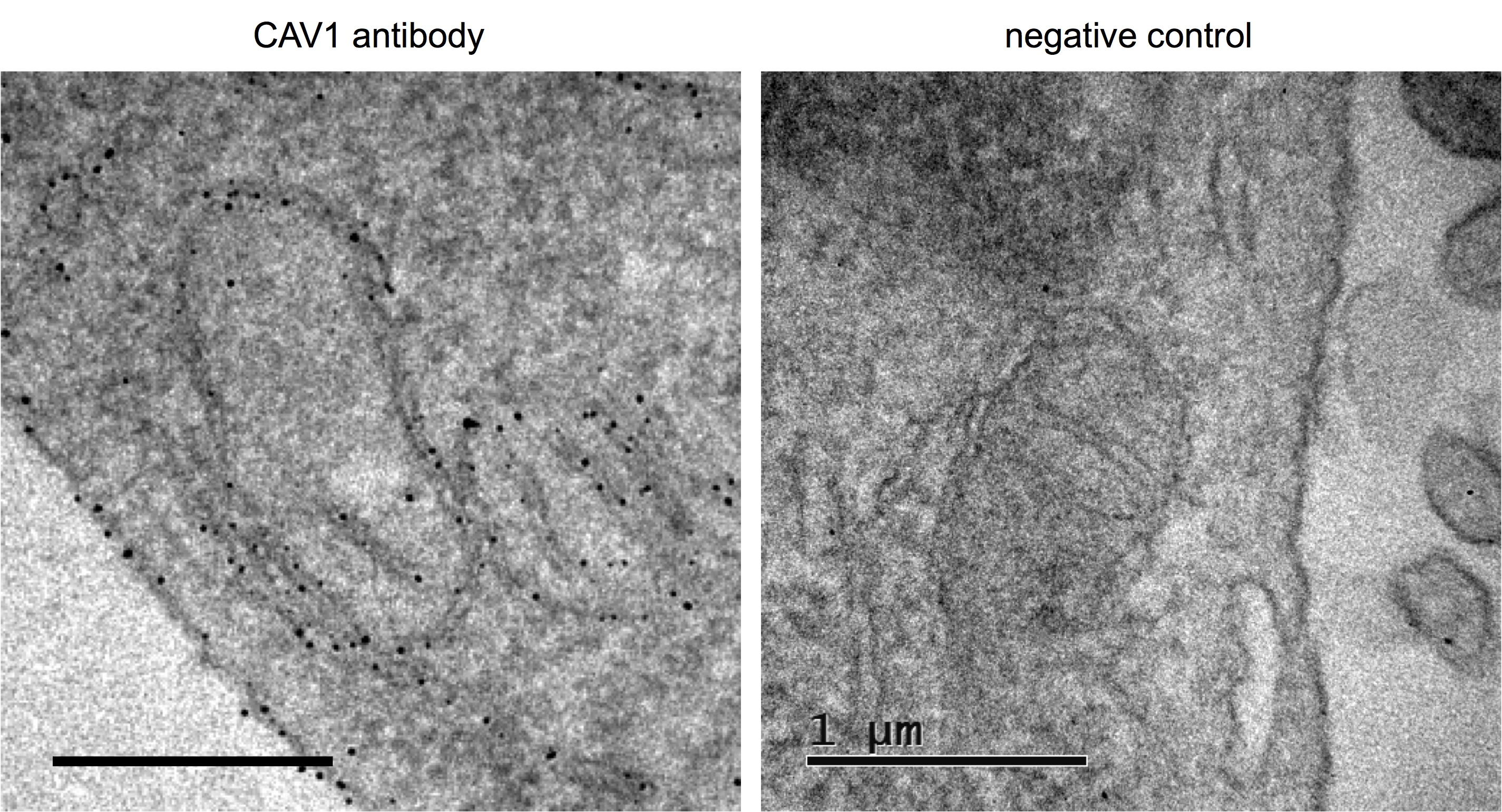

Supplement: Supplementary file 2 — Supplementary figure 2 [file 41418_2018_197_MOESM2_ESM.tif]

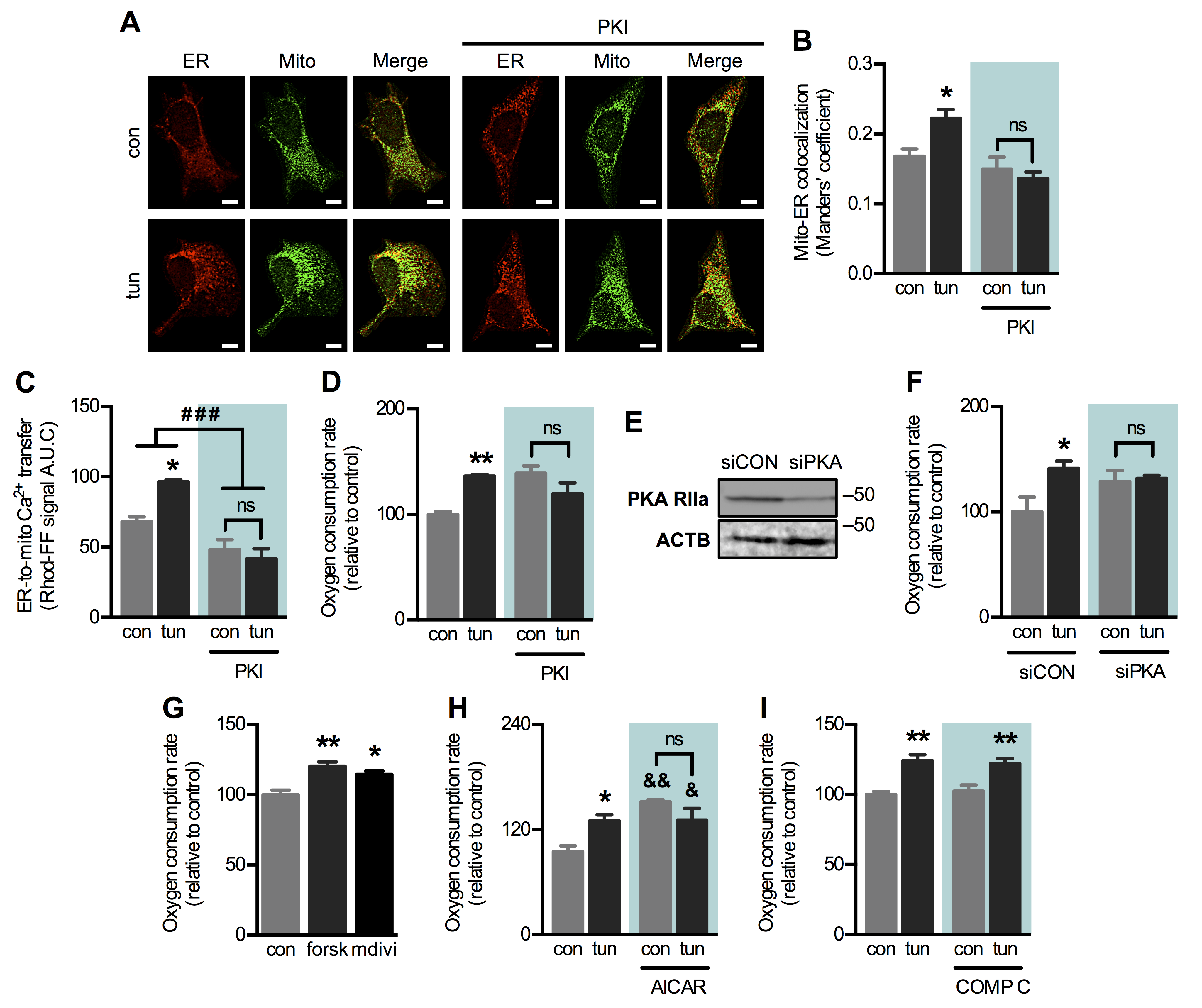

Supplement: Supplementary file 3 — Supplementary figure 3 [file 41418_2018_197_MOESM3_ESM.tif]

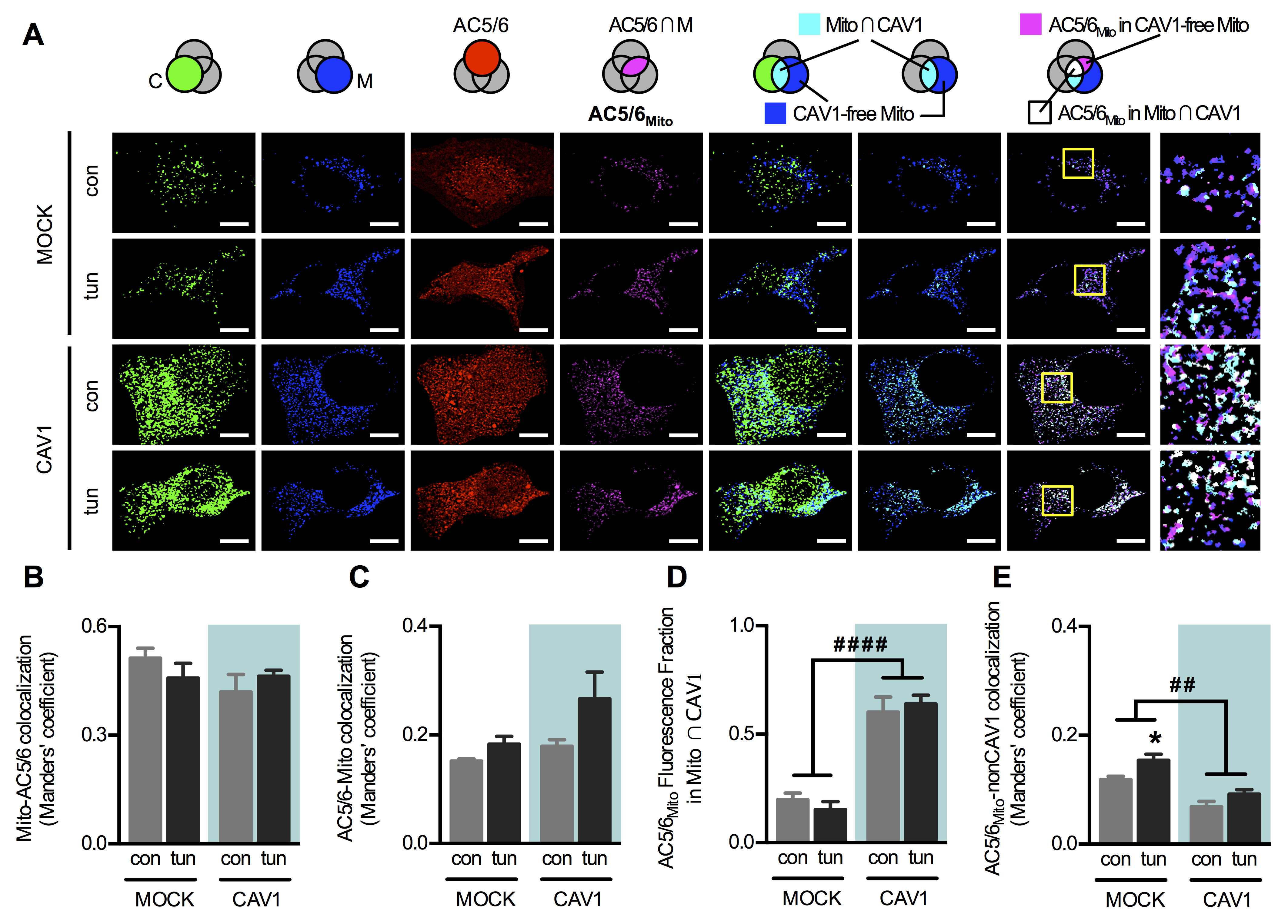

Supplement: Supplementary file 4 — Supplementary figure 4 [file 41418_2018_197_MOESM4_ESM.tif]

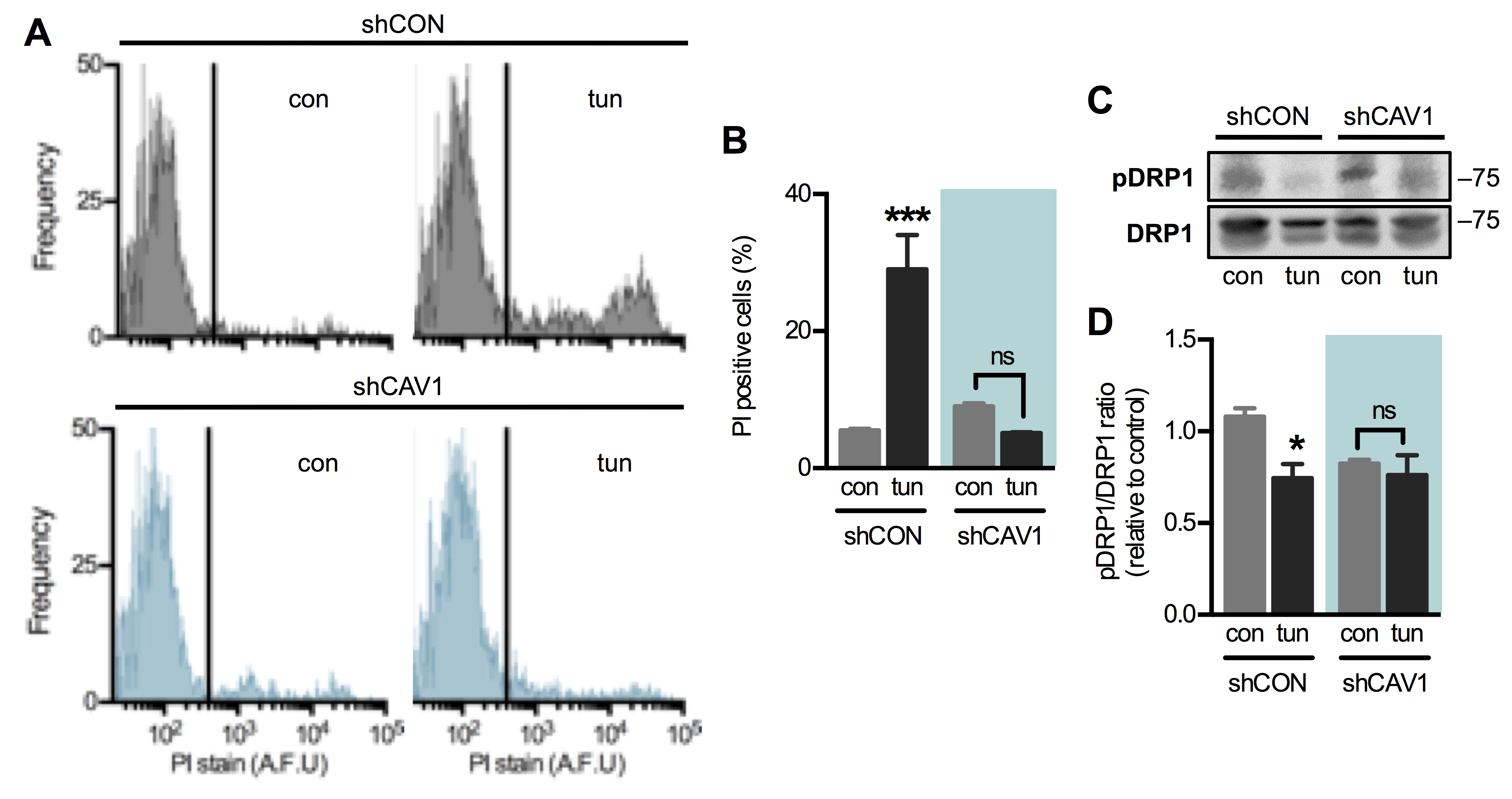

Supplement: Supplementary file 5 — Supplementary figure 5 [file 41418_2018_197_MOESM5_ESM.tif]
