# Supplementary material for: Caveolin-1 impairs PKA-DRP1-mediated remodelling of ER–mitochondria communication during the early phase of ER stress
Source: Cell Death Differ. 2018 Sep 12;26(7):1195–212. doi: 10.1038/s41418-018-0197-1 (PMC6748148; doi:10.1038/s41418-018-0197-1)
Supplement: Supplementary file 6 — supplementary figure legends [file 41418_2018_197_MOESM6_ESM.docx]

# Supplementary figure legends

**Supplementary Figure 1.** CAV1 expression levels in HeLa and MDA-MB-231 cells. (**A**) CAV1 levels of wild-type HeLa cells compared with wild-type MDA-MB-231 cells analysed by western blotting and normalized to β‑actin (ACTB). (**B**) Parental HeLa cells (P) were stably transfected with mock or CAV1‑encoding plasmids. CAV1 and ACTB protein levels were analysed by western blotting. (**C**) HeLa mock and CAV1 cells in control condition (con) or under early ER stress with tunicamycin (tun) were processed, and CAV1 and ACTB protein levels were analysed by western blotting. (**D**) Quantification of CAV1 analysed in C (n = 3). (**E**) MDA-MB-231 cells were stably transfected with shCON or shCAV1 constructs. CAV1 and ACTB levels were analysed by western blotting. (**F**) MDA-MB-231 shCON and shCAV1 cells in control condition (con) or under early ER stress with tunicamycin (tun) were processed, and CAV1 and ACTB protein levels were analysed by western blotting. (**G**) Quantification of CAV1 analysed in F (n = 3). Results are shown as mean ± s.e.m. ^&^P < 0.05, ^&&^P < 0.01 and ^&&&&^P < 0.0001 compared with untreated control cells. ns: non‑significant.

**Supplementary Figure 2.** Immunogold staining of CAV1 in HeLa cells. Wild-type HeLa cells were labelled by immunogold staining with an anti-CAV1 antibody or in the absence of primary antibody (negative control), and then imaged using electron microscopy.

**Supplementary Figure 3.** PKA inhibitors preclude the increase in ER-mitochondria communication during the early phase of ER stress. (**A**) Wild-type HeLa cells in control condition (con) or under early ER stress with tunicamycin (tun) in the absence or presence of PKI were processed for live-cell imaging. The ER was stained with ERTracker Red and mitochondria were stained with MitoTracker Green, and then imaged using confocal microscopy. (**B**) Mitochondria‑ER colocalization was quantified as Manders’ coefficients of images obtained in A (n = 3). (**C**) For experimental groups as in A, Ca^2+^ release from ER stores was induced with histamine, while mitochondrial Ca^2+^ levels were imaged with Rhod-FF using fluorescence microscopy and quantified as the area under the curve (A.U.C.) (n = 3). (**D**) For experimental groups as in A, mitochondrial respiration rates were measured using a Clark electrode (n = 3). (**E**) Wild-type HeLa cells were transfected with siRNA control (siCON) or siRNA against PKA RIIa (siPKA). PKA RIIa levels were analysed by western blotting and normalized to β-actin (ACTB). (**F**) Mitochondrial respiration rates were measured in siCON and siPKA HeLa cells in control condition (con) or under early ER stress with tunicamycin (tun) using a Clark electrode (n = 3). (**G**) Mitochondrial respiration was measured using a Clark electrode in wild-type HeLa cells in control condition (con) or treated with forskolin (forsk) or Mdivi‑1 (mdivi) (n = 3). (**H**) Mitochondrial respiration was measured in wild-type HeLa cells in control condition (con) or under early ER stress with tunicamycin (tun) in the absence or presence of AICAR using a Clark electrode (n = 3). (**I**) Mitochondrial respiration was measured in wild-type HeLa cells in control condition (con) or under early ER stress with tunicamycin (tun) in the absence or presence of compound C (COMP C) using a Clark electrode (n = 3). For each independent imaging experiment, 5‑15 cells were analysed. Scale bars: 10 µm. Results are shown as mean ± s.e.m. *P< 0.05 and **P< 0.01 compared with the respective con conditions. ^####^P < 0.0001 overall comparison between conditions. ^&^P < 0.05 and ^&&^P < 0.01 compared with untreated con cells. ns: non‑significant.

**Supplementary Figure 4.** Caveolin-1 expression alters adenylate cyclase (AC) 5/6 redistribution during early ER stress. (**A**) Mock and CAV1 cells in control condition (con) or under early ER stress with tunicamycin (tun) were processed for immunofluorescence analysis. CAV1, mtHSP70 (mitochondrial marker, Mito) and AC5/6 were stained with the respective antibodies, and then imaged using confocal microscopy. Thresholded CAV1 (C) and Mitochondria (M) images were generated. The intersection between M and AC5/6 reflects mitochondria-associated AC5/6 (AC5/6_Mito_). The merge between M and C images shows colocalized areas in cyan, which correspond to CAV1-containing mitochondrial domains. The merge between Mitochondria, mitochondrial CAV1 and AC5/6_mito_ images shows colocalized pixels in white, which represent areas of triple colocalization. (**B**) Mitochondria‑AC5/6 colocalization was quantified as Manders’ coefficients of images obtained in A (n = 3). (**C**) AC5/6-Mitochondria colocalization was quantified as Manders’ coefficients of images obtained in A (n = 3). (**D**) The fraction of AC5/6_mito_ fluorescence that colocalizes with CAV1-containing mitochondrial domains was quantified from images obtained in A (n = 3). (**E**) The fraction of AC5/6_mito_ fluorescence present in CAV1-free mitochondria was quantified from images obtained in A (n = 3). For each independent imaging experiment, 5‑15 cells were analysed. Scale bars: 10 µm. Results are shown as mean ± s.e.m. *P< 0.05 compared with the respective con conditions. ^##^P < 0.01 and ^####^P < 0.0001 overall comparison between conditions.

**Supplementary Figure 5.** CAV1 silencing in MDA-MB-231 cells protects against ER stress. (**A**) MDA-MB-231 shCON and shCAV1 cells were subjected to the control condition (con) or to cytotoxic ER stress tunicamycin for 24 h (tun)). Cell viability was assessed by propidium iodide (PI) staining using flow cytometry. (**B**) Cell death was quantified as the percentage of PI-positive cells in experiments as in A (n = 3). (**C**) MDA-MB-231 shCON and shCAV1 cells in control condition (con) or under early ER stress with tunicamycin (tun) were analysed by western blotting. DRP1 Ser637 phosphorylation was normalized to total DRP1. (**D**) Quantification of the DRP1 phosphorylation analysed in C (n = 3). In contrast to HeLa cells, DRP1 phosphorylation levels in MDA-MB-231 cells do not correlate with adaptation to ER stress. Instead, they are negatively correlated to the observed changes in Ca^2+^ release from ER stores in these cells (Fig. 2E). Given that the Ca^2+^-dependent phosphatase Calcineurin dephosphorylates DRP1 in Ser637^48^, we propose that the altered Ca^2+^ homeostasis in MDA-MB-231 cells interferes with CAV1/PKA-mediated DRP1 regulation. Results are shown as mean ± s.e.m. *P< 0.05 and ***P< 0.001 compared with the respective con conditions. ns: non‑significant.
